# Epidemiological surveillance of H9N2 avian influenza virus infection among chickens in farms and backyards in Egypt 2015-2016

**DOI:** 10.14202/vetworld.2021.949-955

**Published:** 2021-04-20

**Authors:** Moataz Mohamed El-Sayed, Abdel Satar Arafa, Marwa Abdelmagid, Ahmed Ibrahim Youssef

**Affiliations:** 1 Reference Laboratory for Veterinary Quality Control on Poultry Production, Animal Health Research Institute, P.O. Box 264, Dokki, Giza 12618, Egypt; 2 Animal Hygiene and Zoonoses, Faculty of Veterinary Medicine, Suez Canal University, Ismailia, 41522, Egypt

**Keywords:** Egypt, H9N2 avian influenza virus, influenza, surveillance

## Abstract

**Background and Aim:**

LPAI H9N2 infection among the poultry population in Egypt constitutes an additional risk factor in the poultry industry. This study aimed to determine the prevalence of H9N2 avian influenza virus (AIV) in commercial and backyard chickens in Egypt. A 2-year survey of H9N2 AIV in chickens in farms and backyards was carried out in 2015 and 2016.

**Materials and Methods:**

Direct detection of H9N2 AIV was performed by detecting the virus in tracheal and cloacal swabs using real-time polymerase chain reaction assays. A total of 20,421 samples were collected from chickens in farms and backyards in 26 Egyptian governorates.

**Results:**

In 2015, cases positive for H9N2 AIV numbered 388 (3.9%) out of 10,016 examined cases. However, in 2016, the total positive cases numbered 447 (4.3%) out of 10,405 examined cases. The prevalence of H9N2 AIV among chickens on commercial farms was 4.6% out of the 16,666 chickens examined. The rates of positive cases in 2015 and 2016 were 4.4% (349/7884) and 4.7% (417/8782), respectively. The prevalence of H9N2 AIV in backyard chickens was 1.8% (69/3755). The rates of positive cases in backyard chickens were 1.8% (39/2132) in 2015 and again 1.8% (30/1623) in 2016. The highest positivity rate of H9N2 in chicken farms was in Beni-Suef (61.5%) (8/13), whereas the highest positivity rate in backyard chickens was in Fayoum (8.2%) (8/97).

**Conclusion:**

The analysis of H9N2 infections among chicken farms and in backyard chickens in the different governorates of Egypt over 2 years indicated widespread infection throughout the country. Thus, continuous surveillance and implementation of control programs are warranted.

## Introduction

Since the first detection of H9N2 avian influenza virus (AIV) in poultry species (in turkeys) in the USA in 1966 [1], the virus has spread widely among poultry populations in different countries globally. It has caused substantial economic losses in many countries in Asia and the Middle East [2,3]. A wide range of Avians can act as hosts of H9N2 AIV, including chickens [4], pigeons [5], turkeys, ducks, and geese [6]. Infections of H9N2 AIV have also been recorded in pigs [7]. The low pathogenicity of H9N2 infections among poultry allows the virus to adapt and spread, and also enhances the development of frequent antigenic variations of the circulating virus strains [8].

Zoonotic transmission of H9N2 AIVs have been documented causing mild or asymptomatic infections in humans [9]. The first human case of H9N2 infection was reported in Hong Kong in 1998 [10]. Since then, the virus has continued to infect humans in many countries including Egypt [9,11]. Recently, a human case of H9N2 infection was reported in Oman [12].

Several outbreaks of H9N2 AIV have been documented with the occurrence of single infection and coinfection with other bacterial and viral pathogens [13,14]. The cocirculation of H9 subtypes with other AI subtypes increases the risk of evolution of the circulating influenza virus subtypes to novel subtypes, which could cause a future pandemic [15]. Indeed, the genetic reassortment pattern of H9N2 AIVs might be associated with the genesis of novel zoonotic AIV strains such as H5N1, H7N9, and H5N2 influenza viruses associated with human infection [16-18].

In Egypt, H9N2 AIV was first isolated from a commercial quail farm in 2011 [19]. Since then, the virus has become endemic in different poultry production sectors. A wide geographical spread of H9N2 AIV in both commercial and backyard breeding systems with considerable economic losses has been reported [20,21]. H9N2 virus infection was detected in 20 governorates throughout Egypt from different poultry species, including chickens, turkeys, ducks, and quails [20]. Despite sustained monitoring and control measures, H9N2 AIV continues to circulate in Egypt. A novel reassortant virus from pigeons has been detected [22], and the sharing of genome segments with a novel reassortant HPAI virus H5N2 in broiler chickens have also been reported [18].

The circulation of H9N2 AIV in Egypt increases the need to study the distribution pattern of H9N2 AIV in poultry through extensive surveillance and monitoring of field cases in commercial and backyard chickens throughout the country. Here, surveillance activities were conducted to determine the prevalence of H9N2 AIV in commercial and backyard chickens in Egypt.

## Materials and Methods

### Ethical approval

Ethical approval for this study was obtained from Animal Health Research Institute of Egypt.

### Study location and period

Molecular surveillance of H9N2 AIV was conducted in the Reference Laboratory for Veterinary Quality Control on Poultry Production (RLQP). A 2-year survey of the H9N2 AIV in chicken farms and the household chicken were carried out in 2015 and 2016.

### Sampling

As shown in Table-1, a total of 20,421 samples were collected from chicken farms and backyard chickens (10,016 samples in 2015 and 10,405 samples in 2016). The samples were collected from 26 governorates throughout Egypt representing large four regions: Upper Egypt (Fayoum, Beni-Suef, El-menia, Asuit, Qena, Souhaj, Luxor, Aswan, Al-Wadi Algidid), Lower Egypt (Cairo, Giza, Qaliobia, Menofia, Dakahlia, Gharbia, Sharkia, Kafr El-Sheikh, Behaira), East Egypt (Demiatta, Suez, Ismailia, Port Said, North Sinai, South Sinai), and West Egypt (Alexandria, Matrouh).

**Table-1:** Total incidence of H9N2 AIV infection among farm and backyard chicken in Egypt during 2015-2016.

Year of collection	Breeding sectors				Total	

	Farms		Backyard			
		
	Examined	Positive (%)	Examined	Positive (%)	Examined	Positive (%)
2015	7884	349 (4.4)	2132	39 (1.8)	10016	388 (3.9)
2016	8782	417 (4.7)	1623	30 (1.8)	10405	447 (4.3)
Total	16666	766 (4.6)	3755	69 (1.8)	20421	835 (4.0)

The samples were collected as five tracheal and five cloacal swabs from each collection site. All swabs from each farm or backyard were pooled as a single sample. The swabs were collected in sterile phosphate-buffered saline. Samples were transported under freezing conditions with minimal delay to the Laboratory of Virology, RLQP, Animal Health Research Institute (AHRI), Ministry of Agriculture, Egypt.

### RNA extraction and polymerase chain reaction (PCR) examination

Total RNA was extracted from the pooled samples using a QiaAmp Viral RNA Mini Kit (QIAGEN, Hilden, Germany), in accordance with the manufacturer’s instructions. Extracted RNA was subjected to real-time RT-PCR for detection of the H9 subtype of influenza A virus using Qiagen Quantitect probe real-time RT-PCR kit (Qiagen Inc., Valencia, CA, USA), using a Stratagene Mx3005p real-time PCR system (Agilent Technologies, USA), and the primer sequences and protocol reported previously [23]: H9F primer: 5’-GGAAGAATTAATTATTATTGGTCGGTAC-3’, H9R primer: 5’- GCCACCTTTTTCAGTCTGACAT T-3’, H9 probe: FAM-5’-AACCAGGCCAGACATTGCGAGTAAGATCC-3’ TAMRA.

### Statistical analysis

Differences between groups were analyzed for statistical significance using Chi-squared test with “Statistical Package for Social Sciences, SPSS version 24.0” (IBM Corp, Armonk, NY, USA).

## Results

### Health status of the sampled poultry

On the farms, symptoms including reduced water consumption and respiratory manifestations were observed in broilers, while in layers there was reduced egg production with respiratory distress. At other farms, no apparent symptoms were identified. Mortality reached up to 30%. A few cases were vaccinated against H9 AIV (31 cases), while some cases were vaccinated against H5 AIV (482 cases).

### Total prevalence of H9N2 among sectors and years

As shown in Table-1, in 2015, the total positive cases in farm chickens numbered 388 out of 10,016 examined cases, with a rate of 3.9%. However, in 2016, 447 (4.3%) out of 10,405 examined farm chickens were positive for H9N2 AIV. The prevalence of H9N2 AIV among chickens at commercial farms was estimated at 4.6% (766/16,666). The rates of total positive H9N2 AIV cases in 2015 and 2016 were 4.4% (349/10,016) and 4.7% (417/10,405), respectively. The prevalence of H9N2 AIV in backyard chickens was estimated at 1.8% (69/3755) in 2015, while it was also 1.8% (39/2132) in 2016.

### Prevalence of H9N2 AIV according to governorates

Fayoum Governorate had the highest rates of positive H9N2 detection in chicken farms among the governorates in Upper Egypt, with a 2-year prevalence of 23.6% (34.5% in 2015 and 16.5% in 2016). In contrast, H9N2 infection was not recorded at all in Matrouh Governorate (Table-2). The differences in the positivity rate of H9N2 AIV infections among poultry in all the governorates were non-significant using paired t-test, t(62)=1.57 (p≤ 0.05).

**Table-2:** Tow-year survey of H9N2 AIV infection among different governorates of Egypt in 2015-2016.

Governorates	Year of collection					

	2015		2016		Total	
		
	Examined	Positive (%)	Examined	Positive (%)	Examined	Positive (%)
Giza	441	30 (6.8)	352	23 (6.5)	793	53 (6.9)
Qalubia	694	26 (3.7)	696	40 (5.7)	1390	66 (4.8)
Cairo	45	0 (0)	31	4 (13)	76	4 (5.2)
Monofiya	2299	60 (2.6)	2055	52 (2.5)	4354	112 (2.6)
Dakahlia	1024	43 (4.2)	1389	90 (6.5)	2413	133 (5.5)
Gharbia	147	3 (2.0)	218	7 (3.2)	365	10 (2.7)
Sharkia	1250	14 (1.1)	1023	53 (5.1)	2273	67 (2.9)
Kafr El-Sheikh	80	4 (5)	40	2 (5)	120	6 (5)
Behaira	1147	57 (5)	1011	43 (4.2)	2158	100 (4.6)
Demiatta	48	0 (0)	12	1 (8.3)	60	1 (1.7)
Alexandria	20	0 (0)	23	6 (26)	43	6 (14)
Matrouh	12	0 (0)	2	0 (0)	14	0 (0)
Suez	38	1 (2.6)	37	0 (0)	75	1 (1.3)
Ismailia	185	1 (0.5)	272	7 (2.6)	457	8 (1.8)
Port-Said	13	0 (0)	19	1 (5.3)	32	1 (3.1)
North Sinai	64	3 (4.7)	58	3 (5.1)	122	6 (5)
South Sinai	7	1 (14.2)	2	0 (0)	9	1 (11.1)
Fayoum	116	40 (34.5)	176	29 (16.5)	292	69 (23.6)
Beni-Suef	96	8 (8.3)	103	6 (5.8)	199	14 (7)
El-menia	546	7 (1.3)	1291	23 (1.8)	1837	30 (1.6)
Asuit	862	54 (6.2)	744	34 (4.6)	1606	88 (5.5)
Qena	208	21 (10)	183	7 (3.8)	391	28 (7.1)
Souhaj	127	8 (6.3)	260	9 (3.5)	387	17 (4.4)
Luxor	218	1 (0.4)	149	3 (2)	367	4 (1)
Aswan	17	1 (5.9)	7	0 (0)	24	1 (4.1)
Al Wadi Algidid	312	5 (1.6)	252	4 (1.6)	564	9 1.6)
Total	10016	388 (3.9)	10405	447 (4.3)	20421	835 (4)

### Prevalence of H9N2 AIV according to breeding sectors

**Figure-1:**
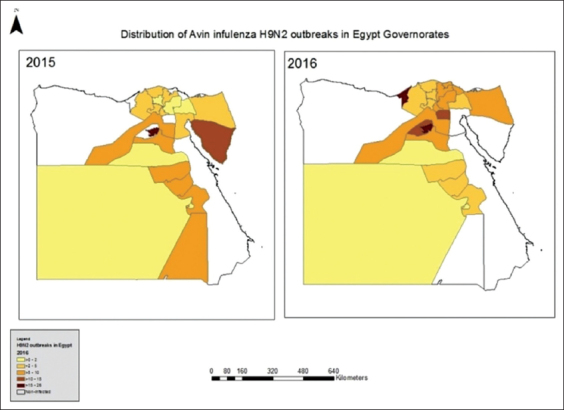
Geographic distribution of H9N2 infections among chicken farms and backyard during 2016 and 2017 throughout the 26 Governorates of Egypt.

The results shown in Table-3 and Figure-1 revealed that, over the 2-year surveillance period, the rate of H9N2 AIV infection was higher in chicken farms (4.6%) than in backyards (1.8%). There was pronounced variation in the prevalence of infection in Beni-Suef Governorate, where the infection rate was 61.5% in farms but 3.2% in backyards. H9N2 infection was not recorded in farms in four governorates (Cairo, Matrouh, South Sinai, and Aswan) and was not recorded in backyards in six governorates (Damietta, Alexandria, Matrouh, Suez, Port Said, and Al-wadi Algadid). Statistical analysis showed that there were no significant differences among the different governorates in the numbers of new cases of H9N2 in chickens. Statistical analysis using paired t-test revealed that the positivity rate of H9N2 AIV infection among farms and backyard chickens was significant (25)=2.85 (p≤ 0.05). There was a correlation between the positivity rate in farms and that in backyards (p≤0.05).

**Table-3:** The prevalence of H9N2 AIV infection in commercial and household chicken among governorates of Egypt (2015-2016).

Governorates	Breeding sectors			

	Farms		Backyards	
	
	Examined	Positive (%)	Examined	Positive (%)
Giza	692	52 (7.5)	101	1 (1)
Qalubia	1250	64 (5.1)	140	2 (1.4)
Cairo	15	0 (0)	61	4 (6.5)
Monofiya	4102	109 (2.7)	252	3 (1.2)
Dakahlia	2050	127 (6.2)	363	6 (1.7)
Gharbia	144	7 (4.9)	221	3 (1.4)
Sharkia	1809	66 (3.6)	464	1 (0.2)
Kafr El-Sheikh	17	5 (29.4)	103	1 (1.0)
Behaira	1752	93 (5.3)	406	7 (1.7)
Demiatta	4	1 (25.0)	56	0 (0)
Alexandria	37	6 (16.2)	6	0 (0)
Matrouh	2	0 (0)	12	0 (0)
Suez	61	1 (1.6)	14	0 (0)
Ismailia	362	5 (1.4)	95	3 (3.1)
Port-Said	17	1 (5.9)	15	0 (0)
North Sinai	100	4 (4)	22	2 (9)
South Sinai	1	0 (0)	8	1 (12.5)
Fayoum	195	61 (31.2)	97	8 (8.2)
Beni-Suef	13	8 (61.5)	186	6 (3.2)
El-menia	1275	23 (1.8)	562	7 (1.2)
Asuit	1556	87 (5.6)	50	1 (2)
Qena	226	22 (9.7)	165	6 (3.6)
Souhaj	126	13 (10.3)	261	4 (1.5)
Luxor	320	2 N(0.6)	47	2 (4.2)
Aswan	5	0(0)	19	1 (5.2)
Al Wadi Algidid	535	9 (1.7)	29	0 (0)
Total	16666	766 (4.6)	3755	69 (1.8)

### Prevalence of H9N2 AIV according to region

In 2015, the prevalence of H9N2 AIV in Upper Egypt (6.9%) was higher than that in Lower Egypt (3.8%), while in 2016, Lower Egypt (5.0%) had a higher positivity rate than Upper Egypt (4.1%). The prevalence of infection in East Egypt was 1.6% in 2015 and 2.6% in 2016 for farms and it was 1.8% in 2015 and 4.1% in 2016 for backyards. The infection was not detected in West Egypt in backyards in 2015**-**2016, while it was detected in farms in this region only in 2016 (Table-4). Statistical analysis using Chi-square showed that there were significant differences between the geographic regions of Egypt (p= 0.01).

**Table-4:** Geo-incidence of H9N2 virus among farm and backyard chicken in different districts of Egypt (2015-2016).

Geographical area (District)	Farms				Backyards				
	
	2015		2016		2015			2016	
			
	Examined	Positive (%)	Examined	Positive (%)	Examined	Positive (%)		Examined	Positive (%)
Upper	1792	124 (6.9)	2459	101 (4.1)	710	21	(3)	706	14 (2)
Lower	5834	221 (3.8)	5997	302 (5)	1293	16	(1.2)	818	12 (1.5)
East	241	4 (1.6)	304	8 (2.6)	114	2	(1.8)	96	4 (4.1)
West	17	0 (0)	22	6 (27.2)	15	0	(0)	3	0 (0)

### Number of new cases per month over the 2 years

**Figure-2:**
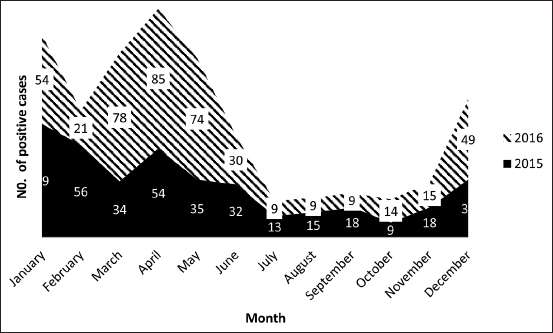
Monthly distribution of positive H9N2 avian influenza virus cases during 2015-2016.

As shown in Figure-2, in 2015, the highest numbers of cases occurred in January, February, and April with 69, 56, and 54 cases, whereas the fewest cases occurred in October, July, and August with 9, 13, and 15 cases, respectively. In 2016, the highest recorded numbers of cases were in April, March, and May, respectively, with 85, 78, and 74 cases, while the fewest cases were 9 in each of July, August, and September.

## Discussion

The endemicity and widespread of H9N2 AIV infection among the poultry population in Egypt have made it an additional risk factor in the poultry industry. H9N2 AIV also has the potential to be transmitted to humans, especially with the cocirculation of other influenza subtypes in Egypt [18,24]. Available epidemiological data revealed that H9N2 AIV was initially distributed through Lower Egypt and Giza Governorate. Subsequently, H9N2 viruses were reported to have become widespread in most governorates, including in Upper Egypt, with H9N2 infections found among quails and chickens [25]. H9N2 AIV infection was recorded at a higher incidence among apparently healthy broilers than among layers [20,25].

In this study, H9N2 AIV was surveyed among chickens in commercial farms and backyards in 26 governorates throughout Egypt. The data on the geographical prevalence of H9 subtype-positive cases revealed that the infection was recorded in 21/26 governorates in 2015 and 22/26 governorates in 2016. The spread of the virus showed no particular geographical pattern as there were no distinct barriers between most of the Egyptian governorates. In 2015, H9N2 infection was recorded in Upper Egypt, Lower Egypt, and East Egypt, while it was absent in West Egypt. Meanwhile, in 2016, the infection was recorded in all the four regions of Egypt. These results confirm the endemicity of H9N2 AIV infection among chicken populations in Egypt. Our results showed that the total prevalence of chicken infections with H9N2 was 3.9% in 2015 and 4.3% in 2016, which was higher than that reported in the previous work (3%) [25]. The distribution of poultry breeding is associated with population density and the urban/rural divide among different governorates in Egypt. It was reported that governorates in Middle Egypt (Giza, Fayoum, and Bani-Sweef) are major centers in the LPAIV H9N2 transmission network in the country [26]. The differences in the prevalence of H9N2 AIV infection among governorates and regions might be associated with the density of chickens and the movement of birds between cities and governorates. In addition, the number of samples that were collected from each governorate might affect the prevalence of infection, as there were low numbers of samples in some governorates.

Our results revealed that H9 infection was recorded in almost all governorates included in this study during 2015 and 2016, with the exception of Matrouh. This geographically widespread of H9N2 AIV infection in chickens in Egypt might be attributable to several factors, such as the low pathogenicity of the virus, poor biosecurity measures, and lack of a scheduled vaccination program. Moreover, household breeding is common in Egypt, where the birds are raised in a free-range set-up or at homes without biosecurity measures or vaccinations. Besides, the birds in backyards are regularly in close contact with carrier species such as aquatic birds, and with free-flying birds such as migratory birds, pigeons, and quails, which could be infected or act as carriers for different influenza subtypes [27]. Other factors associated with the widespread of H9N2 AIV infection among poultry in Egypt are wild bird and live bird markets. In another study, H9N2 influenza virus was detected in surveillance of AIV in wild birds at live bird markets in Egypt in the same period as when the current study was performed [28].

The results of this surveillance indicated that the positivity rate in the commercial poultry sector (4.6%) was higher than that in the household poultry sector (1.8%), which matches the findings in previous work documenting a positivity rate in the commercial sector of 3.3%, but one in the backyard sector of 0.74% [25]. In addition, it was previously reported by Arafa *et al*. [20] that the prevalence of H9 infection among commercial poultry was higher than that in backyards. The detectable circulation of H9N2 AIV in commercial chickens may indicate some deficiencies in applying vaccinations and biosecurity strategies, representing a risk to the poultry industry, especially with the presence of disease-associated infections with other pathogens or other variants of AIVs.

The prevention and control programs of influenza viruses are focused on H5 AIVs subtype. However, the significance and economic impacts of H9N2 AIV should be considered when establishing programs to prevent and control H9 AIVs subtype. Although our findings indicated that the spread of H9N2 AIV among backyard birds occurred at a low rate, the backyard system is considered a major problem as it provides the opportunity for more poultry species to be reared together. In Egypt, particularly in rural regions, chickens, ducks, geese, turkeys, and pigeons are often reared in close contact with other animals and humans in the same household. The birds are mostly reared in primitive rooftops, cages, or as scavengers without the application of biosecurity measures. They move or graze through the streets or fields. These birds may thus come into contact with either domestic poultry or migratory birds [29]. Thus, the inter-species and intra-species transmission of viruses could occur. As a consequence, new genotypes of H9N2 viruses might emerge, such as those previously reported to contain genes of marine avian origin as a result of two-way transmission between terrestrial and aquatic birds [17,30]. In addition, vaccinating household birds are difficult as most owners of backyard poultry show little or no cooperation with the authorities. All of these factors make household birds a potential reservoir and mixing vessel for the selection of AIV variants.

In this study, the incidence of H9N2 infection increased during the winter and spring months, while it decreased throughout the summer and fall, when the temperature increased. The climatic patterns of H9N2 infection in 2015 and 2016 were similar, with the infection rate gradually decreasing from April to June, and then infection being detected at a low rate from July to November. Finally, the infection rate increased from December to April. An increase in the rate of positive H9N2 cases in the spring months indicated that the epidemiology of H9N2 in chickens in Egypt in 2015 and 2016 changed over time, with outbreaks now occurring in warmer months. In 2015, the highest recorded numbers of cases were in January, February, and April, while in 2016 they were in April, March, and May. The results of this study agree with the previous work that reported H9N2 AIV infection throughout the year, with a particular association with winter months in Egypt during 2011**-**2012 [21]. In addition, our results agreed with a previous study [31] that found that AIV H9N2 infection increased during the winter in Northern Pakistan, but during the summer in Southern Pakistan. The detection of infection all year round confirms the endemic nature of this infection in Egypt.

Avian influenza of the H9N2 subtype could be naturally transmitted and humans contacts with infected birds [10]. Seropositivity for the H9N2 virus was also reported in those who work with poultry [11]. Moreover, the continuous prevalence of the H9N2 virus in the Middle East has generated a much fitter or optimized replication phenotype, leading to an expanded viral host range, including mammals. This may pose public health risks beyond those associated with the outbreaks that have occurred to date [32]. H9N2 virus has a risk for cross-species transmission, as it circulates at the avian**-**human interface and contributes to the emergence of new reassortants, which could potentially produce a pandemic [11]. Therefore, cocirculation of H5N1 and H9N2 viruses in the poultry industry and live bird markets increases the risk of human exposure, resulting in growing concerns about the potential emergence of a new influenza virus pandemic [33].

H9N2 AIVs are continuing to circulate through inter-species and intra-species transmission among various host species and subsequent zoonotic transmission has been reported by Rahimi Rad *et al*. [11]. Genetic and antigenic changes in the virus, host range, virus evolution, climatic pattern, and geographical spread should be continuously monitored. Without the implementation of effective strategies including proper biosecurity measures, development of strategic vaccination programs, as well as establishing national and international collaboration, H9N2 AIV infections are likely to continue to impact the poultry industry in Egypt.

## Conclusion

The high prevalence of H9N2 infections among chicken farms and backyards in Egypt over 2 years indicated that such infections are widespread throughout the country. Thus, there is a need to encourage adequate measures to control H9N2 AIV in poultry in Egypt to minimize the risk factors to the poultry industry and the possibility of novel pandemic variants arising.

## Authors’ Contributions

MME drafted the manuscript, AIY revised the manuscript, analyzed the data, ASA designated the work, MME and MA collected the samples, and performed the laboratory work. All authors read and approved the final manuscript.
